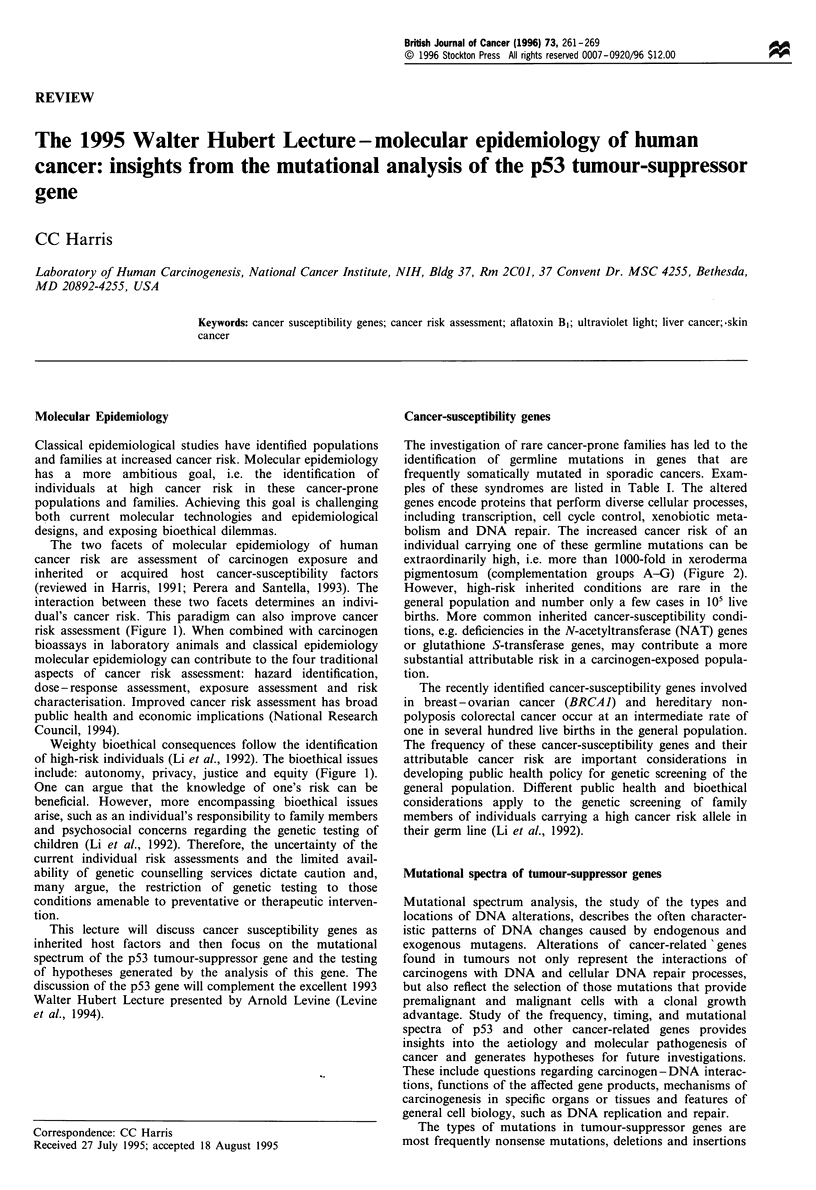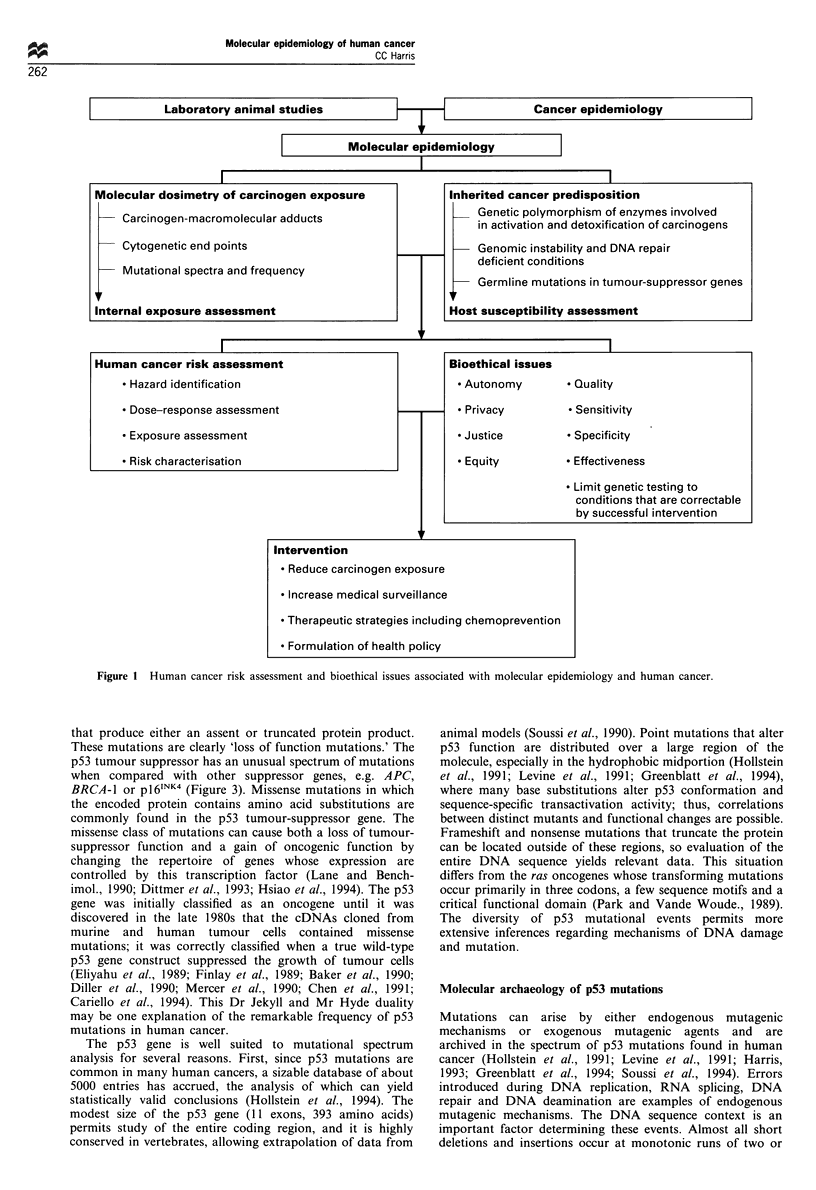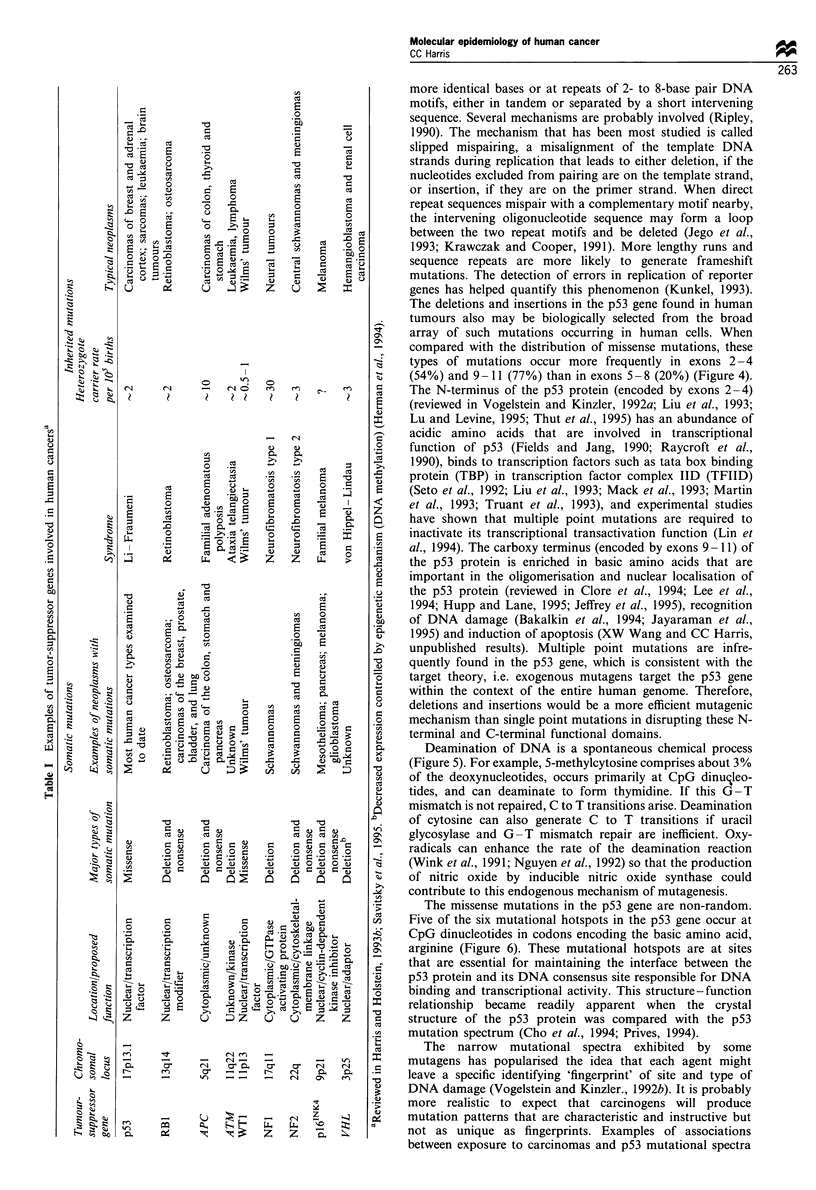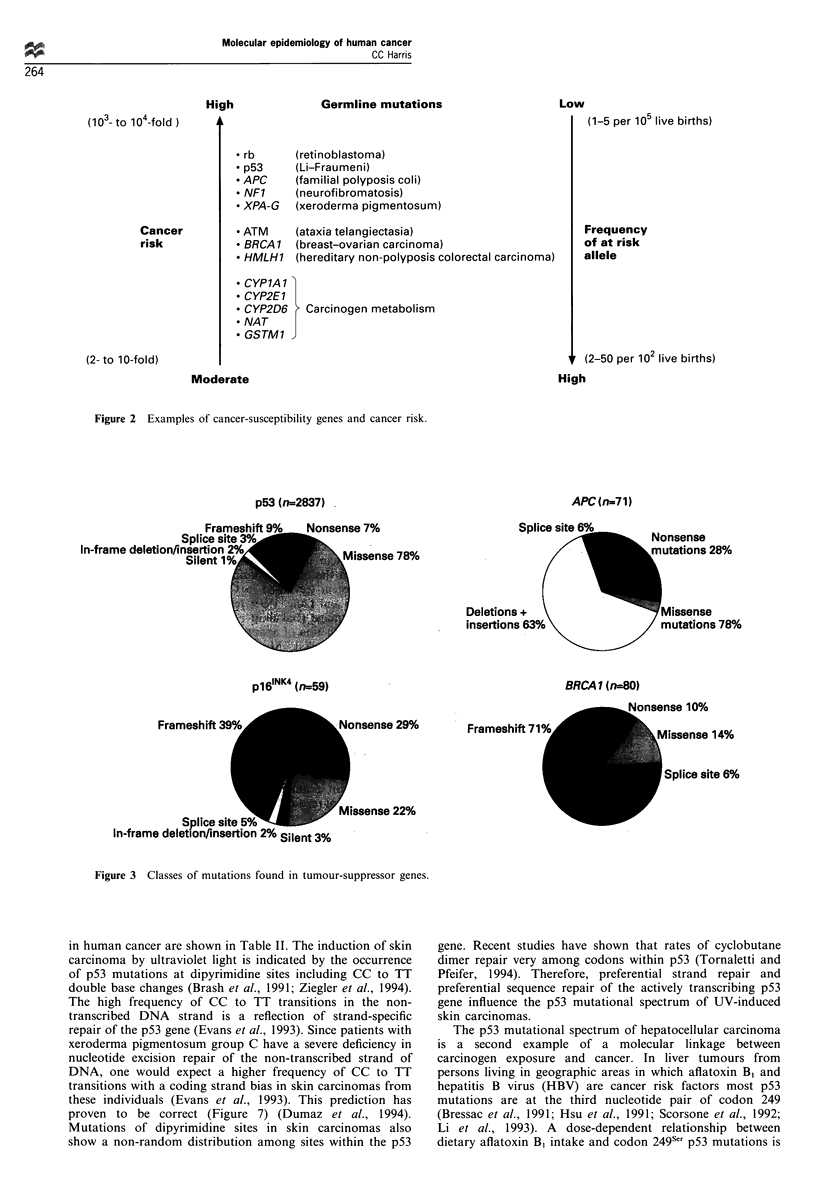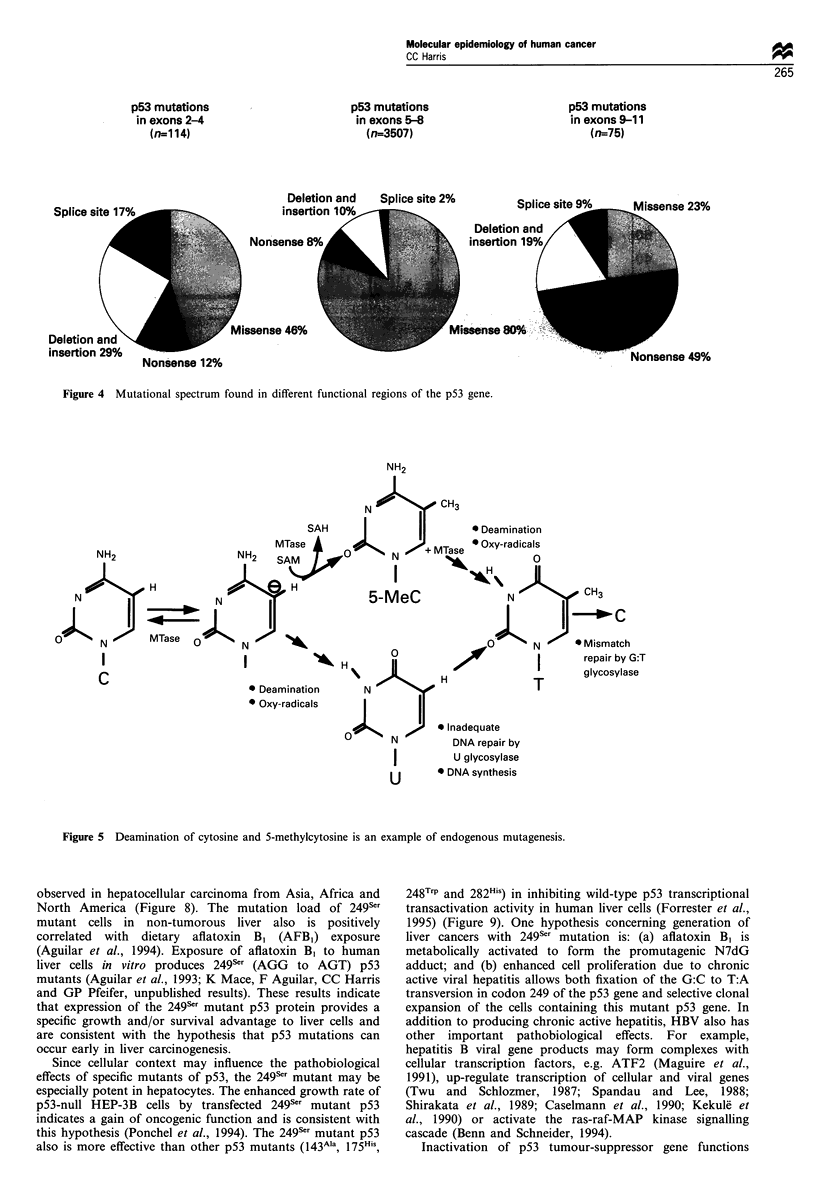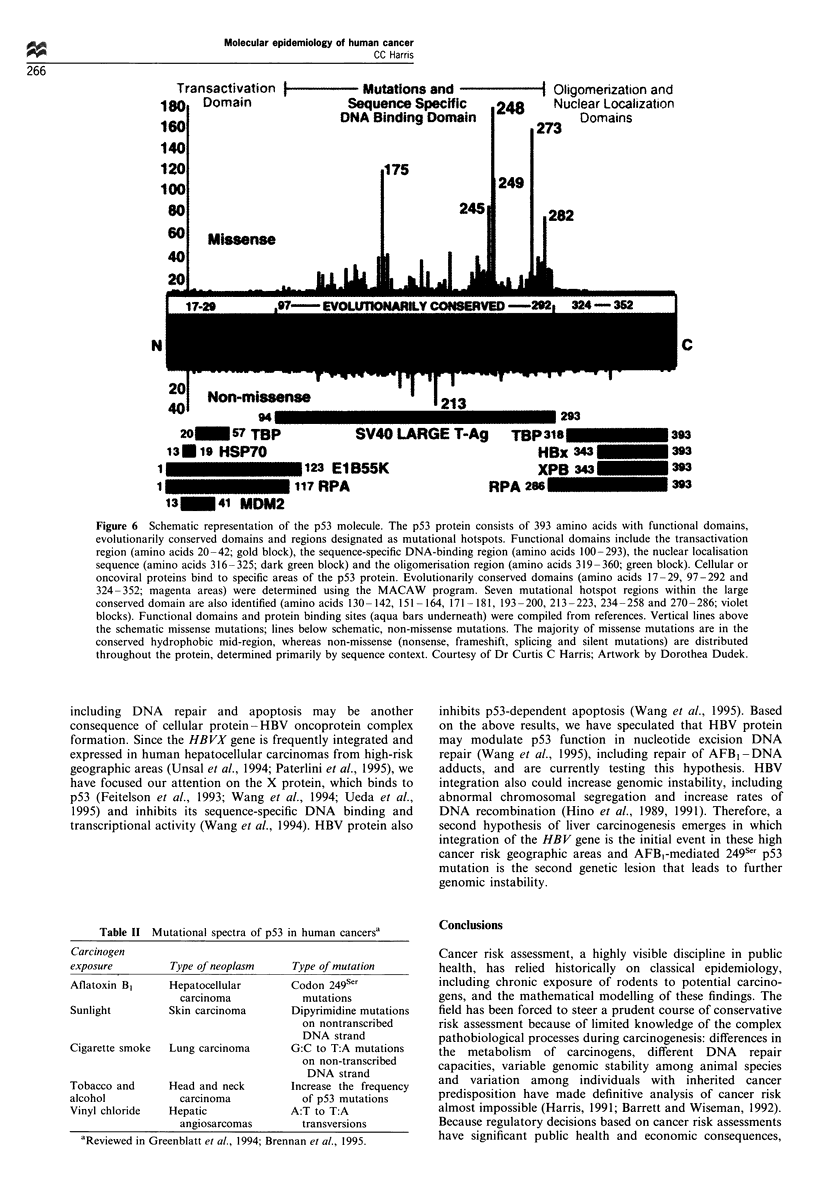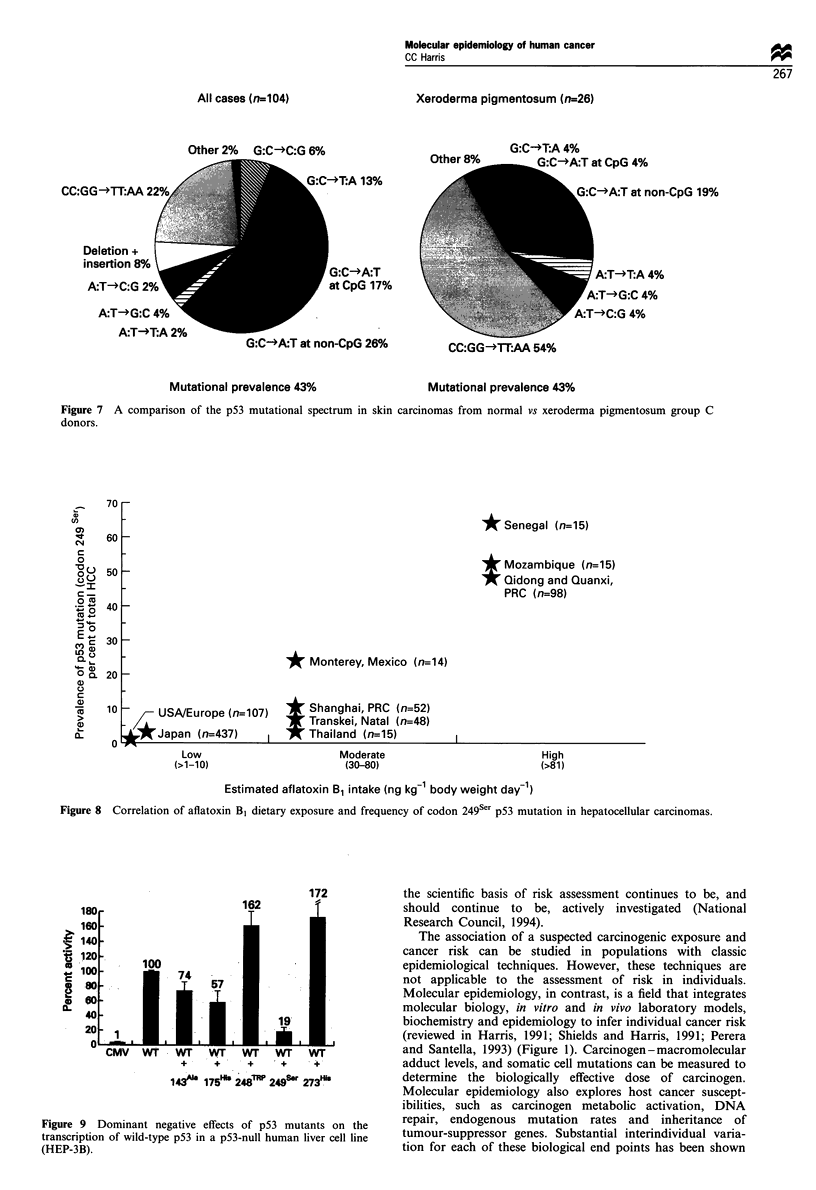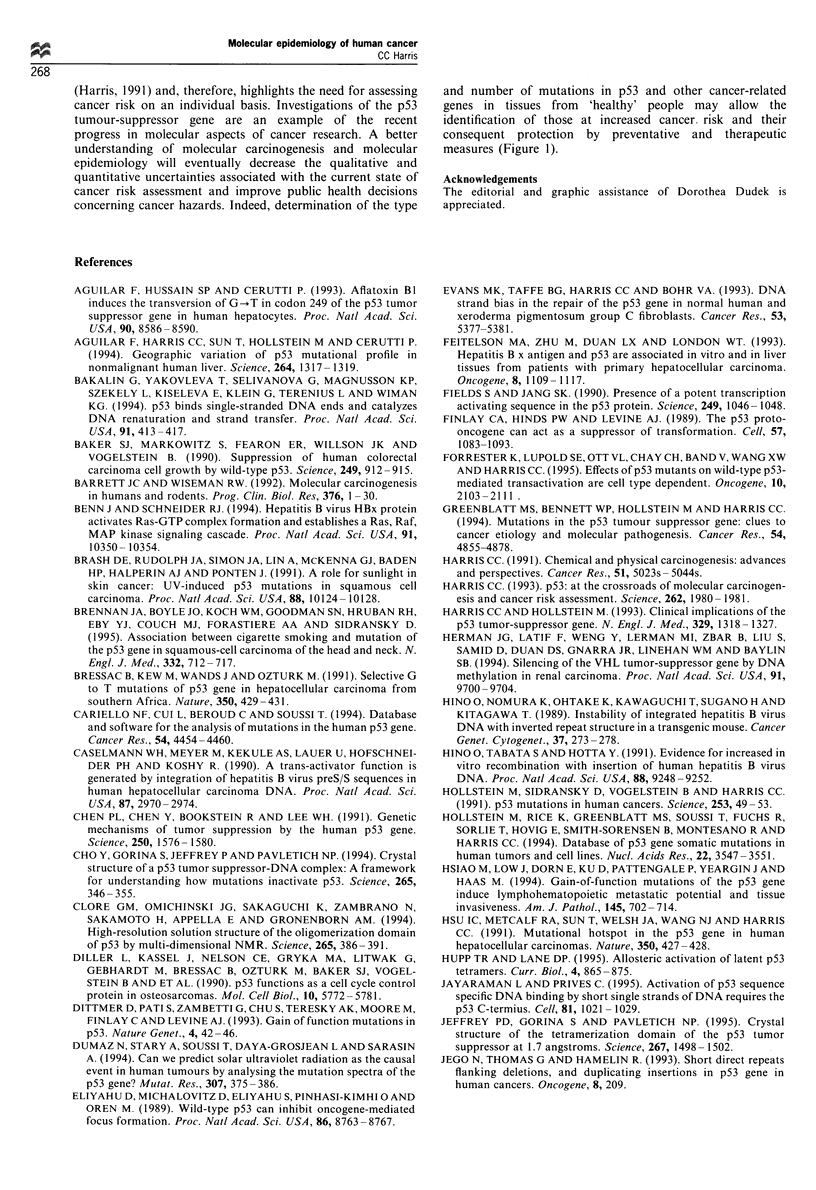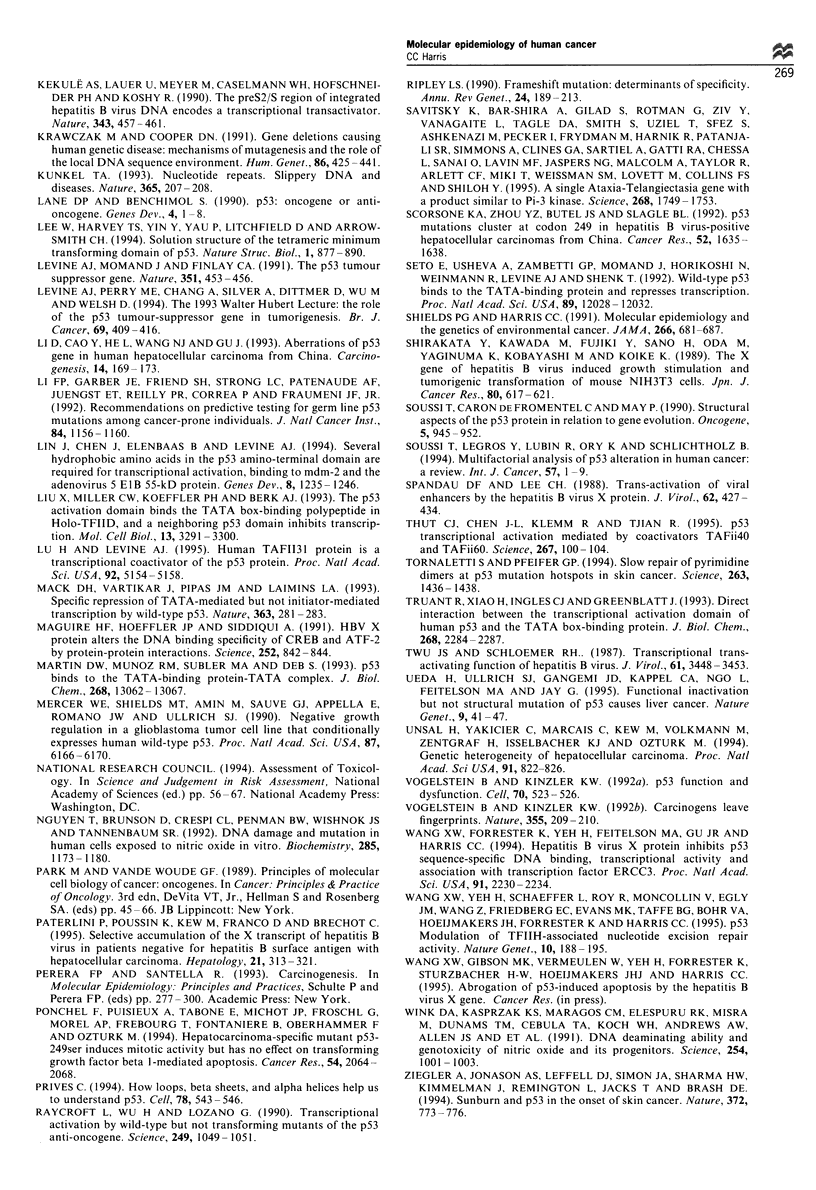# The 1995 Walter Hubert Lecture--molecular epidemiology of human cancer: insights from the mutational analysis of the p53 tumour-suppressor gene.

**DOI:** 10.1038/bjc.1996.47

**Published:** 1996-02

**Authors:** C. C. Harris

**Affiliations:** Laboratory of Human Carcinogenesis, National Cancer Institute, NIH, Bethesda, MD 20892-4255, USA.

## Abstract

**Images:**


British Journal of Cancer (1996) 73, 261-269
© 1996 Stockton Press All rights reserved 0007-0920/96 $12.00

REVIEW

### The 1995 Walter Hubert Lecture – molecular epidemiology of human cancer: insights from the mutational analysis of the p53 tumour-suppressor gene

CC Harris

*Laboratory of Human Carcinogenesis, National Cancer Institute, NIH, Bldg 37, Rm 2C01, 37 Convent Dr. MSC 4255, Bethesda, MD 20892-4255, USA*

**Keywords:** cancer susceptibility genes; cancer risk assessment; aflatoxin $B_1$; ultraviolet light; liver cancer; skin cancer

### Molecular Epidemiology

Classical epidemiological studies have identified populations and families at increased cancer risk. Molecular epidemiology has a more ambitious goal, i.e. the identification of individuals at high cancer risk in these cancer-prone populations and families. Achieving this goal is challenging both current molecular technologies and epidemiological designs, and exposing bioethical dilemmas.

The two facets of molecular epidemiology of human cancer risk are assessment of carcinogen exposure and inherited or acquired host cancer-susceptibility factors (reviewed in Harris, 1991; Perera and Santella, 1993). The interaction between these two facets determines an individual's cancer risk. This paradigm can also improve cancer risk assessment (Figure 1). When combined with carcinogen bioassays in laboratory animals and classical epidemiology molecular epidemiology can contribute to the four traditional aspects of cancer risk assessment: hazard identification, dose–response assessment, exposure assessment and risk characterisation. Improved cancer risk assessment has broad public health and economic implications (National Research Council, 1994).

Weighty bioethical consequences follow the identification of high-risk individuals (Li *et al.*, 1992). The bioethical issues include: autonomy, privacy, justice and equity (Figure 1). One can argue that the knowledge of one's risk can be beneficial. However, more encompassing bioethical issues arise, such as an individual's responsibility to family members and psychosocial concerns regarding the genetic testing of children (Li *et al.*, 1992). Therefore, the uncertainty of the current individual risk assessments and the limited availability of genetic counselling services dictate caution and, many argue, the restriction of genetic testing to those conditions amenable to preventative or therapeutic intervention.

This lecture will discuss cancer susceptibility genes as inherited host factors and then focus on the mutational spectrum of the p53 tumour-suppressor gene and the testing of hypotheses generated by the analysis of this gene. The discussion of the p53 gene will complement the excellent 1993 Walter Hubert Lecture presented by Arnold Levine (Levine *et al.*, 1994).

Correspondence: CC Harris
Received 27 July 1995; accepted 18 August 1995

### Cancer-susceptibility genes

The investigation of rare cancer-prone families has led to the identification of germline mutations in genes that are frequently somatically mutated in sporadic cancers. Examples of these syndromes are listed in Table I. The altered genes encode proteins that perform diverse cellular processes, including transcription, cell cycle control, xenobiotic metabolism and DNA repair. The increased cancer risk of an individual carrying one of these germline mutations can be extraordinarily high, i.e. more than 1000-fold in xeroderma pigmentosum (complementation groups A–G) (Figure 2). However, high-risk inherited conditions are rare in the general population and number only a few cases in $10^5$ live births. More common inherited cancer-susceptibility conditions, e.g. deficiencies in the *N*-acetyltransferase (NAT) genes or glutathione *S*-transferase genes, may contribute a more substantial attributable risk in a carcinogen-exposed population.

The recently identified cancer-susceptibility genes involved in breast–ovarian cancer (*BRCA1*) and hereditary non-polyposis colorectal cancer occur at an intermediate rate of one in several hundred live births in the general population. The frequency of these cancer-susceptibility genes and their attributable cancer risk are important considerations in developing public health policy for genetic screening of the general population. Different public health and bioethical considerations apply to the genetic screening of family members of individuals carrying a high cancer risk allele in their germ line (Li *et al.*, 1992).

### Mutational spectra of tumour-suppressor genes

Mutational spectrum analysis, the study of the types and locations of DNA alterations, describes the often characteristic patterns of DNA changes caused by endogenous and exogenous mutagens. Alterations of cancer-related genes found in tumours not only represent the interactions of carcinogens with DNA and cellular DNA repair processes, but also reflect the selection of those mutations that provide premalignant and malignant cells with a clonal growth advantage. Study of the frequency, timing, and mutational spectra of p53 and other cancer-related genes provides insights into the aetiology and molecular pathogenesis of cancer and generates hypotheses for future investigations. These include questions regarding carcinogen–DNA interactions, functions of the affected gene products, mechanisms of carcinogenesis in specific organs or tissues and features of general cell biology, such as DNA replication and repair.

The types of mutations in tumour-suppressor genes are most frequently nonsense mutations, deletions and insertions

**Figure 1** Human cancer risk assessment and bioethical issues associated with molecular epidemiology and human cancer.

that produce either an assent or truncated protein product. These mutations are clearly 'loss of function mutations.' The p53 tumour suppressor has an unusual spectrum of mutations when compared with other suppressor genes, e.g. *APC*, *BRCA*-1 or p16$^{INK4}$ (Figure 3). Missense mutations in which the encoded protein contains amino acid substitutions are commonly found in the p53 tumour-suppressor gene. The missense class of mutations can cause both a loss of tumour-suppressor function and a gain of oncogenic function by changing the repertoire of genes whose expression are controlled by this transcription factor (Lane and Benchimol., 1990; Dittmer *et al.*, 1993; Hsiao *et al.*, 1994). The p53 gene was initially classified as an oncogene until it was discovered in the late 1980s that the cDNAs cloned from murine and human tumour cells contained missense mutations; it was correctly classified when a true wild-type p53 gene construct suppressed the growth of tumour cells (Eliyahu *et al.*, 1989; Finlay *et al.*, 1989; Baker *et al.*, 1990; Diller *et al.*, 1990; Mercer *et al.*, 1990; Chen *et al.*, 1991; Cariello *et al.*, 1994). This Dr Jekyll and Mr Hyde duality may be one explanation of the remarkable frequency of p53 mutations in human cancer.

The p53 gene is well suited to mutational spectrum analysis for several reasons. First, since p53 mutations are common in many human cancers, a sizable database of about 5000 entries has accrued, the analysis of which can yield statistically valid conclusions (Hollstein *et al.*, 1994). The modest size of the p53 gene (11 exons, 393 amino acids) permits study of the entire coding region, and it is highly conserved in vertebrates, allowing extrapolation of data from animal models (Soussi *et al.*, 1990). Point mutations that alter p53 function are distributed over a large region of the molecule, especially in the hydrophobic midportion (Hollstein *et al.*, 1991; Levine *et al.*, 1991; Greenblatt *et al.*, 1994), where many base substitutions alter p53 conformation and sequence-specific transactivation activity; thus, correlations between distinct mutants and functional changes are possible. Frameshift and nonsense mutations that truncate the protein can be located outside of these regions, so evaluation of the entire DNA sequence yields relevant data. This situation differs from the *ras* oncogenes whose transforming mutations occur primarily in three codons, a few sequence motifs and a critical functional domain (Park and Vande Woude., 1989). The diversity of p53 mutational events permits more extensive inferences regarding mechanisms of DNA damage and mutation.

### Molecular archaeology of p53 mutations

Mutations can arise by either endogenous mutagenic mechanisms or exogenous mutagenic agents and are archived in the spectrum of p53 mutations found in human cancer (Hollstein *et al.*, 1991; Levine *et al.*, 1991; Harris, 1993; Greenblatt *et al.*, 1994; Soussi *et al.*, 1994). Errors introduced during DNA replication, RNA splicing, DNA repair and DNA deamination are examples of endogenous mutagenic mechanisms. The DNA sequence context is an important factor determining these events. Almost all short deletions and insertions occur at monotonic runs of two or

**Table I** Examples of tumor-suppressor genes involved in human cancers[a]

| Tumour-suppressor gene | Chromosomal locus | Location/proposed function | Major types of somatic mutation | Somatic mutations: Examples of neoplasms with somatic mutations | Inherited mutations: Syndrome | Heterozygote carrier rate per $10^5$ births | Typical neoplasms |
|---|---|---|---|---|---|---|---|
| p53 | 17p13.1 | Nuclear/transcription factor | Missense | Most human cancer types examined to date | Li-Fraumeni | ~2 | Carcinomas of breast and adrenal cortex; sarcomas; leukaemia; brain tumours |
| RB1 | 13q14 | Nuclear/transcription modifier | Deletion and nonsense | Retinoblastoma; osteosarcoma; carcinomas of the breast, prostate, bladder, and lung | Retinoblastoma | ~2 | Retinoblastoma; osteosarcoma |
| *APC* | 5q21 | Cytoplasmic/unknown | Deletion and nonsense | Carcinoma of the colon, stomach and pancreas | Familial adenomatous polyposis | ~10 | Carcinomas of colon, thyroid and stomach |
| *ATM* | 11q22 | Unknown/kinase | Deletion | Unknown | Ataxia telangiectasia | ~2 | Leukaemia, lymphoma |
| WT1 | 11p13 | Nuclear/transcription factor | Missense | Wilms' tumour | Wilms' tumour | ~0.5-1 | Wilms' tumour |
| NF1 | 17q11 | Cytoplasmic/GTPase activating protein | Deletion | Schwannomas | Neurofibromatosis type 1 | ~30 | Neural tumours |
| NF2 | 22q | Cytoplasmic/cytoskeletal-membrane linkage | Deletion and nonsense | Schwannomas and meningiomas | Neurofibromatosis type 2 | ~3 | Central schwannomas and meningiomas |
| p16$^{INK4}$ | 9p21 | Nuclear/cyclin-dependent kinase inhibitor | Deletion and nonsense | Mesothelioma; pancreas; melanoma; glioblastoma | Familial melanoma | ? | Melanoma |
| *VHL* | 3p25 | Nuclear/adaptor | Deletion[b] | Unknown | von Hippel-Lindau | ~3 | Hemangioblastoma and renal cell carcinoma |

[a]Reviewed in Harris and Holstein, 1993*b*; Savitsky *et al*., 1995. [b]Decreased expression controlled by epigenetic mechanism (DNA methylation) (Herman *et al*., 1994).

more identical bases or at repeats of 2- to 8-base pair DNA motifs, either in tandem or separated by a short intervening sequence. Several mechanisms are probably involved (Ripley, 1990). The mechanism that has been most studied is called slipped mispairing, a misalignment of the template DNA strands during replication that leads to either deletion, if the nucleotides excluded from pairing are on the template strand, or insertion, if they are on the primer strand. When direct repeat sequences mispair with a complementary motif nearby, the intervening oligonucleotide sequence may form a loop between the two repeat motifs and be deleted (Jego *et al*., 1993; Krawczak and Cooper, 1991). More lengthy runs and sequence repeats are more likely to generate frameshift mutations. The detection of errors in replication of reporter genes has helped quantify this phenomenon (Kunkel, 1993). The deletions and insertions in the p53 gene found in human tumours also may be biologically selected from the broad array of such mutations occurring in human cells. When compared with the distribution of missense mutations, these types of mutations occur more frequently in exons 2-4 (54%) and 9-11 (77%) than in exons 5-8 (20%) (Figure 4). The N-terminus of the p53 protein (encoded by exons 2-4) (reviewed in Vogelstein and Kinzler, 1992*a*; Liu *et al*., 1993; Lu and Levine, 1995; Thut *et al*., 1995) has an abundance of acidic amino acids that are involved in transcriptional function of p53 (Fields and Jang, 1990; Raycroft *et al*., 1990), binds to transcription factors such as tata box binding protein (TBP) in transcription factor complex IID (TFIID) (Seto *et al*., 1992; Liu *et al*., 1993; Mack *et al*., 1993; Martin *et al*., 1993; Truant *et al*., 1993), and experimental studies have shown that multiple point mutations are required to inactivate its transcriptional transactivation function (Lin *et al*., 1994). The carboxy terminus (encoded by exons 9-11) of the p53 protein is enriched in basic amino acids that are important in the oligomerisation and nuclear localisation of the p53 protein (reviewed in Clore *et al*., 1994; Lee *et al*., 1994; Hupp and Lane, 1995; Jeffrey *et al*., 1995), recognition of DNA damage (Bakalkin *et al*., 1994; Jayaraman *et al*., 1995) and induction of apoptosis (XW Wang and CC Harris, unpublished results). Multiple point mutations are infrequently found in the p53 gene, which is consistent with the target theory, i.e. exogenous mutagens target the p53 gene within the context of the entire human genome. Therefore, deletions and insertions would be a more efficient mutagenic mechanism than single point mutations in disrupting these N-terminal and C-terminal functional domains.

Deamination of DNA is a spontaneous chemical process (Figure 5). For example, 5-methylcytosine comprises about 3% of the deoxynucleotides, occurs primarily at CpG dinucleotides, and can deaminate to form thymidine. If this G-T mismatch is not repaired, C to T transitions arise. Deamination of cytosine can also generate C to T transitions if uracil glycosylase and G-T mismatch repair are inefficient. Oxyradicals can enhance the rate of the deamination reaction (Wink *et al*., 1991; Nguyen *et al*., 1992) so that the production of nitric oxide by inducible nitric oxide synthase could contribute to this endogenous mechanism of mutagenesis.

The missense mutations in the p53 gene are non-random. Five of the six mutational hotspots in the p53 gene occur at CpG dinucleotides in codons encoding the basic amino acid, arginine (Figure 6). These mutational hotspots are at sites that are essential for maintaining the interface between the p53 protein and its DNA consensus site responsible for DNA binding and transcriptional activity. This structure-function relationship became readily apparent when the crystal structure of the p53 protein was compared with the p53 mutation spectrum (Cho *et al*., 1994; Prives, 1994).

The narrow mutational spectra exhibited by some mutagens has popularised the idea that each agent might leave a specific identifying 'fingerprint' of site and type of DNA damage (Vogelstein and Kinzler., 1992*b*). It is probably more realistic to expect that carcinogens will produce mutation patterns that are characteristic and instructive but not as unique as fingerprints. Examples of associations between exposure to carcinomas and p53 mutational spectra

**Figure 2** Examples of cancer-susceptibility genes and cancer risk.

**Figure 3** Classes of mutations found in tumour-suppressor genes.

in human cancer are shown in Table II. The induction of skin carcinoma by ultraviolet light is indicated by the occurrence of p53 mutations at dipyrimidine sites including CC to TT double base changes (Brash *et al.*, 1991; Ziegler *et al.*, 1994). The high frequency of CC to TT transitions in the non-transcribed DNA strand is a reflection of strand-specific repair of the p53 gene (Evans *et al.*, 1993). Since patients with xeroderma pigmentosum group C have a severe deficiency in nucleotide excision repair of the non-transcribed strand of DNA, one would expect a higher frequency of CC to TT transitions with a coding strand bias in skin carcinomas from these individuals (Evans *et al.*, 1993). This prediction has proven to be correct (Figure 7) (Dumaz *et al.*, 1994). Mutations of dipyrimidine sites in skin carcinomas also show a non-random distribution among sites within the p53 gene. Recent studies have shown that rates of cyclobutane dimer repair very among codons within p53 (Tornaletti and Pfeifer, 1994). Therefore, preferential strand repair and preferential sequence repair of the actively transcribing p53 gene influence the p53 mutational spectrum of UV-induced skin carcinomas.

The p53 mutational spectrum of hepatocellular carcinoma is a second example of a molecular linkage between carcinogen exposure and cancer. In liver tumours from persons living in geographic areas in which aflatoxin $B_1$ and hepatitis B virus (HBV) are cancer risk factors most p53 mutations are at the third nucleotide pair of codon 249 (Bressac *et al.*, 1991; Hsu *et al.*, 1991; Scorsone *et al.*, 1992; Li *et al.*, 1993). A dose-dependent relationship between dietary aflatoxin $B_1$ intake and codon $249^{Ser}$ p53 mutations is

**Figure 4** Mutational spectrum found in different functional regions of the p53 gene.

**Figure 5** Deamination of cytosine and 5-methylcytosine is an example of endogenous mutagenesis.

observed in hepatocellular carcinoma from Asia, Africa and North America (Figure 8). The mutation load of $249^{Ser}$ mutant cells in non-tumorous liver also is positively correlated with dietary aflatoxin $B_1$ ($AFB_1$) exposure (Aguilar *et al.*, 1994). Exposure of aflatoxin $B_1$ to human liver cells *in vitro* produces $249^{Ser}$ (AGG to AGT) p53 mutants (Aguilar *et al.*, 1993; K Mace, F Aguilar, CC Harris and GP Pfeifer, unpublished results). These results indicate that expression of the $249^{Ser}$ mutant p53 protein provides a specific growth and/or survival advantage to liver cells and are consistent with the hypothesis that p53 mutations can occur early in liver carcinogenesis.

Since cellular context may influence the pathobiological effects of specific mutants of p53, the $249^{Ser}$ mutant may be especially potent in hepatocytes. The enhanced growth rate of p53-null HEP-3B cells by transfected $249^{Ser}$ mutant p53 indicates a gain of oncogenic function and is consistent with this hypothesis (Ponchel *et al.*, 1994). The $249^{Ser}$ mutant p53 also is more effective than other p53 mutants ($143^{Ala}$, $175^{His}$, $248^{Trp}$ and $282^{His}$) in inhibiting wild-type p53 transcriptional transactivation activity in human liver cells (Forrester *et al.*, 1995) (Figure 9). One hypothesis concerning generation of liver cancers with $249^{Ser}$ mutation is: (a) aflatoxin $B_1$ is metabolically activated to form the promutagenic N7dG adduct; and (b) enhanced cell proliferation due to chronic active viral hepatitis allows both fixation of the G:C to T:A transversion in codon 249 of the p53 gene and selective clonal expansion of the cells containing this mutant p53 gene. In addition to producing chronic active hepatitis, HBV also has other important pathobiological effects. For example, hepatitis B viral gene products may form complexes with cellular transcription factors, e.g. ATF2 (Maguire *et al.*, 1991), up-regulate transcription of cellular and viral genes (Twu and Schlozmer, 1987; Spandau and Lee, 1988; Shirakata *et al.*, 1989; Caselmann *et al.*, 1990; Kekulë *et al.*, 1990) or activate the ras-raf-MAP kinase signalling cascade (Benn and Schneider, 1994).

Inactivation of p53 tumour-suppressor gene functions

**Figure 6** Schematic representation of the p53 molecule. The p53 protein consists of 393 amino acids with functional domains, evolutionarily conserved domains and regions designated as mutational hotspots. Functional domains include the transactivation region (amino acids 20–42; gold block), the sequence-specific DNA-binding region (amino acids 100–293), the nuclear localisation sequence (amino acids 316–325; dark green block) and the oligomerisation region (amino acids 319–360; green block). Cellular or oncoviral proteins bind to specific areas of the p53 protein. Evolutionarily conserved domains (amino acids 17–29, 97–292 and 324–352; magenta areas) were determined using the MACAW program. Seven mutational hotspot regions within the large conserved domain are also identified (amino acids 130–142, 151–164, 171–181, 193–200, 213–223, 234–258 and 270–286; violet blocks). Functional domains and protein binding sites (aqua bars underneath) were compiled from references. Vertical lines above the schematic missense mutations; lines below schematic, non-missense mutations. The majority of missense mutations are in the conserved hydrophobic mid-region, whereas non-missense (nonsense, frameshift, splicing and silent mutations) are distributed throughout the protein, determined primarily by sequence context. Courtesy of Dr Curtis C Harris; Artwork by Dorothea Dudek.

including DNA repair and apoptosis may be another consequence of cellular protein–HBV oncoprotein complex formation. Since the *HBVX* gene is frequently integrated and expressed in human hepatocellular carcinomas from high-risk geographic areas (Unsal *et al*., 1994; Paterlini *et al*., 1995), we have focused our attention on the X protein, which binds to p53 (Feitelson *et al*., 1993; Wang *et al*., 1994; Ueda *et al*., 1995) and inhibits its sequence-specific DNA binding and transcriptional activity (Wang *et al*., 1994). HBV protein also inhibits p53-dependent apoptosis (Wang *et al*., 1995). Based on the above results, we have speculated that HBV protein may modulate p53 function in nucleotide excision DNA repair (Wang *et al*., 1995), including repair of $AFB_1$–DNA adducts, and are currently testing this hypothesis. HBV integration also could increase genomic instability, including abnormal chromosomal segregation and increase rates of DNA recombination (Hino *et al*., 1989, 1991). Therefore, a second hypothesis of liver carcinogenesis emerges in which integration of the *HBV* gene is the initial event in these high cancer risk geographic areas and $AFB_1$-mediated $249^{Ser}$ p53 mutation is the second genetic lesion that leads to further genomic instability.

**Table II** Mutational spectra of p53 in human cancers[a]

| *Carcinogen exposure* | *Type of neoplasm* | *Type of mutation* |
|---|---|---|
| Aflatoxin $B_1$ | Hepatocellular carcinoma | Codon $249^{Ser}$ mutations |
| Sunlight | Skin carcinoma | Dipyrimidine mutations on nontranscribed DNA strand |
| Cigarette smoke | Lung carcinoma | G:C to T:A mutations on non-transcribed DNA strand |
| Tobacco and alcohol | Head and neck carcinoma | Increase the frequency of p53 mutations |
| Vinyl chloride | Hepatic angiosarcomas | A:T to T:A transversions |

[a]Reviewed in Greenblatt *et al*., 1994; Brennan *et al*., 1995.

### Conclusions

Cancer risk assessment, a highly visible discipline in public health, has relied historically on classical epidemiology, including chronic exposure of rodents to potential carcinogens, and the mathematical modelling of these findings. The field has been forced to steer a prudent course of conservative risk assessment because of limited knowledge of the complex pathobiological processes during carcinogenesis: differences in the metabolism of carcinogens, different DNA repair capacities, variable genomic stability among animal species and variation among individuals with inherited cancer predisposition have made definitive analysis of cancer risk almost impossible (Harris, 1991; Barrett and Wiseman, 1992). Because regulatory decisions based on cancer risk assessments have significant public health and economic consequences,

**Figure 7** A comparison of the p53 mutational spectrum in skin carcinomas from normal *vs* xeroderma pigmentosum group C donors.

**Figure 8** Correlation of aflatoxin $B_1$ dietary exposure and frequency of codon $249^{Ser}$ p53 mutation in hepatocellular carcinomas.

**Figure 9** Dominant negative effects of p53 mutants on the transcription of wild-type p53 in a p53-null human liver cell line (HEP-3B).

the scientific basis of risk assessment continues to be, and should continue to be, actively investigated (National Research Council, 1994).

The association of a suspected carcinogenic exposure and cancer risk can be studied in populations with classic epidemiological techniques. However, these techniques are not applicable to the assessment of risk in individuals. Molecular epidemiology, in contrast, is a field that integrates molecular biology, *in vitro* and *in vivo* laboratory models, biochemistry and epidemiology to infer individual cancer risk (reviewed in Harris, 1991; Shields and Harris, 1991; Perera and Santella, 1993) (Figure 1). Carcinogen–macromolecular adduct levels, and somatic cell mutations can be measured to determine the biologically effective dose of carcinogen. Molecular epidemiology also explores host cancer susceptibilities, such as carcinogen metabolic activation, DNA repair, endogenous mutation rates and inheritance of tumour-suppressor genes. Substantial interindividual variation for each of these biological end points has been shown

(Harris, 1991) and, therefore, highlights the need for assessing cancer risk on an individual basis. Investigations of the p53 tumour-suppressor gene are an example of the recent progress in molecular aspects of cancer research. A better understanding of molecular carcinogenesis and molecular epidemiology will eventually decrease the qualitative and quantitative uncertainties associated with the current state of cancer risk assessment and improve public health decisions concerning cancer hazards. Indeed, determination of the type and number of mutations in p53 and other cancer-related genes in tissues from 'healthy' people may allow the identification of those at increased cancer. risk and their consequent protection by preventative and therapeutic measures (Figure 1).

**Acknowledgements**
The editorial and graphic assistance of Dorothea Dudek is appreciated.

### References

AGUILAR F, HUSSAIN SP AND CERUTTI P. (1993). Aflatoxin B1 induces the transversion of G→T in codon 249 of the p53 tumor suppressor gene in human hepatocytes. *Proc. Natl Acad. Sci. USA*, **90**, 8586-8590.

AGUILAR F, HARRIS CC, SUN T, HOLLSTEIN M AND CERUTTI P. (1994). Geographic variation of p53 mutational profile in nonmalignant human liver. *Science*, **264**, 1317-1319.

BAKALIN G, YAKOVLEVA T, SELIVANOVA G, MAGNUSSON KP, SZEKELY L, KISELEVA E, KLEIN G, TERENIUS L AND WIMAN KG. (1994). p53 binds single-stranded DNA ends and catalyzes DNA renaturation and strand transfer. *Proc. Natl Acad. Sci. USA*, **91**, 413-417.

BAKER SJ, MARKOWITZ S, FEARON ER, WILLSON JK AND VOGELSTEIN B. (1990). Suppression of human colorectal carcinoma cell growth by wild-type p53. *Science*, **249**, 912-915.

BARRETT JC AND WISEMAN RW. (1992). Molecular carcinogenesis in humans and rodents. *Prog. Clin. Biol. Res*, **376**, 1-30.

BENN J AND SCHNEIDER RJ. (1994). Hepatitis B virus HBx protein activates Ras-GTP complex formation and establishes a Ras, Raf, MAP kinase signaling cascade. *Proc. Natl Acad. Sci. USA*, **91**, 10350-10354.

BRASH DE, RUDOLPH JA, SIMON JA, LIN A, McKENNA GJ, BADEN HP, HALPERIN AJ AND PONTEN J. (1991). A role for sunlight in skin cancer: UV-induced p53 mutations in squamous cell carcinoma. *Proc. Natl Acad. Sci. USA*, **88**, 10124-10128.

BRENNAN JA, BOYLE JO, KOCH WM, GOODMAN SN, HRUBAN RH, EBY YJ, COUCH MJ, FORASTIERE AA AND SIDRANSKY D. (1995). Association between cigarette smoking and mutation of the p53 gene in squamous-cell carcinoma of the head and neck. *N. Engl. J. Med.*, **332**, 712-717.

BRESSAC B, KEW M, WANDS J AND OZTURK M. (1991). Selective G to T mutations of p53 gene in hepatocellular carcinoma from southern Africa. *Nature*, **350**, 429-431.

CARIELLO NF, CUI L, BEROUD C AND SOUSSI T. (1994). Database and software for the analysis of mutations in the human p53 gene. *Cancer Res.*, **54**, 4454-4460.

CASELMANN WH, MEYER M, KEKULE AS, LAUER U, HOFSCHNEIDER PH AND KOSHY R. (1990). A trans-activator function is generated by integration of hepatitis B virus preS/S sequences in human hepatocellular carcinoma DNA. *Proc. Natl Acad. Sci. USA*, **87**, 2970-2974.

CHEN PL, CHEN Y, BOOKSTEIN R AND LEE WH. (1991). Genetic mechanisms of tumor suppression by the human p53 gene. *Science*, **250**, 1576-1580.

CHO Y, GORINA S, JEFFREY P AND PAVLETICH NP. (1994). Crystal structure of a p53 tumor suppressor-DNA complex: A framework for understanding how mutations inactivate p53. *Science*, **265**, 346-355.

CLORE GM, OMICHINSKI JG, SAKAGUCHI K, ZAMBRANO N, SAKAMOTO H, APPELLA E AND GRONENBORN AM. (1994). High-resolution solution structure of the oligomerization domain of p53 by multi-dimensional NMR. *Science*, **265**, 386-391.

DILLER L, KASSEL J, NELSON CE, GRYKA MA, LITWAK G, GEBHARDT M, BRESSAC B, OZTURK M, BAKER SJ, VOGELSTEIN B AND ET AL. (1990). p53 functions as a cell cycle control protein in osteosarcomas. *Mol. Cell Biol.*, **10**, 5772-5781.

DITTMER D, PATI S, ZAMBETTI G, CHU S, TERESKY AK, MOORE M, FINLAY C AND LEVINE AJ. (1993). Gain of function mutations in p53. *Nature Genet.*, **4**, 42-46.

DUMAZ N, STARY A, SOUSSI T, DAYA-GROSJEAN L AND SARASIN A. (1994). Can we predict solar ultraviolet radiation as the causal event in human tumours by analysing the mutation spectra of the p53 gene? *Mutat. Res.*, **307**, 375-386.

ELIYAHU D, MICHALOVITZ D, ELIYAHU S, PINHASI-KIMHI O AND OREN M. (1989). Wild-type p53 can inhibit oncogene-mediated focus formation. *Proc. Natl Acad. Sci. USA*, **86**, 8763-8767.

EVANS MK, TAFFE BG, HARRIS CC AND BOHR VA. (1993). DNA strand bias in the repair of the p53 gene in normal human and xeroderma pigmentosum group C fibroblasts. *Cancer Res.*, **53**, 5377-5381.

FEITELSON MA, ZHU M, DUAN LX AND LONDON WT. (1993). Hepatitis B x antigen and p53 are associated in vitro and in liver tissues from patients with primary hepatocellular carcinoma. *Oncogene*, **8**, 1109-1117.

FIELDS S AND JANG SK. (1990). Presence of a potent transcription activating sequence in the p53 protein. *Science*, **249**, 1046-1048.

FINLAY CA, HINDS PW AND LEVINE AJ. (1989). The p53 proto-oncogene can act as a suppressor of transformation. *Cell*, **57**, 1083-1093.

FORRESTER K, LUPOLD SE, OTT VL, CHAY CH, BAND V, WANG XW AND HARRIS CC. (1995). Effects of p53 mutants on wild-type p53-mediated transactivation are cell type dependent. *Oncogene*, **10**, 2103-2111 .

GREENBLATT MS, BENNETT WP, HOLLSTEIN M AND HARRIS CC. (1994). Mutations in the p53 tumour suppressor gene: clues to cancer etiology and molecular pathogenesis. *Cancer Res.*, **54**, 4855-4878.

HARRIS CC. (1991). Chemical and physical carcinogenesis: advances and perspectives. *Cancer Res.*, **51**, 5023s-5044s.

HARRIS CC. (1993). p53: at the crossroads of molecular carcinogenesis and cancer risk assessment. *Science*, **262**, 1980-1981.

HARRIS CC AND HOLLSTEIN M. (1993). Clinical implications of the p53 tumor-suppressor gene. *N. Engl. J. Med.*, **329**, 1318-1327.

HERMAN JG, LATIF F, WENG Y, LERMAN MI, ZBAR B, LIU S, SAMID D, DUAN DS, GNARRA JR, LINEHAN WM AND BAYLIN SB. (1994). Silencing of the VHL tumor-suppressor gene by DNA methylation in renal carcinoma. *Proc. Natl Acad. Sci. USA*, **91**, 9700-9704.

HINO O, NOMURA K, OHTAKE K, KAWAGUCHI T, SUGANO H AND KITAGAWA T. (1989). Instability of integrated hepatitis B virus DNA with inverted repeat structure in a transgenic mouse. *Cancer Genet. Cytogenet.*, **37**, 273-278.

HINO O, TABATA S AND HOTTA Y. (1991). Evidence for increased in vitro recombination with insertion of human hepatitis B virus DNA. *Proc. Natl Acad. Sci. USA*, **88**, 9248-9252.

HOLLSTEIN M, SIDRANSKY D, VOGELSTEIN B AND HARRIS CC. (1991). p53 mutations in human cancers. *Science*, **253**, 49-53.

HOLLSTEIN M, RICE K, GREENBLATT MS, SOUSSI T, FUCHS R, SORLIE T, HOVIG E, SMITH-SORENSEN B, MONTESANO R AND HARRIS CC. (1994). Database of p53 gene somatic mutations in human tumors and cell lines. *Nucl. Acids Res.*, **22**, 3547-3551.

HSIAO M, LOW J, DORN E, KU D, PATTENGALE P, YEARGIN J AND HAAS M. (1994). Gain-of-function mutations of the p53 gene induce lymphohematopoietic metastatic potential and tissue invasiveness. *Am. J. Pathol.*, **145**, 702-714.

HSU IC, METCALF RA, SUN T, WELSH JA, WANG NJ AND HARRIS CC. (1991). Mutational hotspot in the p53 gene in human hepatocellular carcinomas. *Nature*, **350**, 427-428.

HUPP TR AND LANE DP. (1995). Allosteric activation of latent p53 tetramers. *Curr. Biol.*, **4**, 865-875.

JAYARAMAN L AND PRIVES C. (1995). Activation of p53 sequence specific DNA binding by short single strands of DNA requires the p53 C-termius. *Cell*, **81**, 1021-1029.

JEFFREY PD, GORINA S AND PAVLETICH NP. (1995). Crystal structure of the tetramerization domain of the p53 tumor suppressor at 1.7 angstroms. *Science*, **267**, 1498-1502.

JEGO N, THOMAS G AND HAMELIN R. (1993). Short direct repeats flanking deletions, and duplicating insertions in p53 gene in human cancers. *Oncogene*, **8**, 209.

KEKULË AS, LAUER U, MEYER M, CASELMANN WH, HOFSCHNEIDER PH AND KOSHY R. (1990). The preS2/S region of integrated hepatitis B virus DNA encodes a transcriptional transactivator. *Nature*, **343**, 457–461.

KRAWCZAK M AND COOPER DN. (1991). Gene deletions causing human genetic disease: mechanisms of mutagenesis and the role of the local DNA sequence environment. *Hum. Genet.*, **86**, 425–441.

KUNKEL TA. (1993). Nucleotide repeats. Slippery DNA and diseases. *Nature*, **365**, 207–208.

LANE DP AND BENCHIMOL S. (1990). p53: oncogene or anti-oncogene. *Genes Dev.*, **4**, 1–8.

LEE W, HARVEY TS, YIN Y, YAU P, LITCHFIELD D AND ARROWSMITH CH. (1994). Solution structure of the tetrameric minimum transforming domain of p53. *Nature Struc. Biol.*, **1**, 877–890.

LEVINE AJ, MOMAND J AND FINLAY CA. (1991). The p53 tumour suppressor gene. *Nature*, **351**, 453–456.

LEVINE AJ, PERRY ME, CHANG A, SILVER A, DITTMER D, WU M AND WELSH D. (1994). The 1993 Walter Hubert Lecture: the role of the p53 tumour-suppressor gene in tumorigenesis. *Br. J. Cancer*, **69**, 409–416.

LI D, CAO Y, HE L, WANG NJ AND GU J. (1993). Aberrations of p53 gene in human hepatocellular carcinoma from China. *Carcinogenesis*, **14**, 169–173.

LI FP, GARBER JE, FRIEND SH, STRONG LC, PATENAUDE AF, JUENGST ET, REILLY PR, CORREA P AND FRAUMENI JF, JR. (1992). Recommendations on predictive testing for germ line p53 mutations among cancer-prone individuals. *J. Natl Cancer Inst.*, **84**, 1156–1160.

LIN J, CHEN J, ELENBAAS B AND LEVINE AJ. (1994). Several hydrophobic amino acids in the p53 amino-terminal domain are required for transcriptional activation, binding to mdm-2 and the adenovirus 5 E1B 55-kD protein. *Genes Dev.*, **8**, 1235–1246.

LIU X, MILLER CW, KOEFFLER PH AND BERK AJ. (1993). The p53 activation domain binds the TATA box-binding polypeptide in Holo-TFIID, and a neighboring p53 domain inhibits transcription. *Mol. Cell Biol.*, **13**, 3291–3300.

LU H AND LEVINE AJ. (1995). Human TAFII31 protein is a transcriptional coactivator of the p53 protein. *Proc. Natl Acad. Sci. USA*, **92**, 5154–5158.

MACK DH, VARTIKAR J, PIPAS JM AND LAIMINS LA. (1993). Specific repression of TATA-mediated but not initiator-mediated transcription by wild-type p53. *Nature*, **363**, 281–283.

MAGUIRE HF, HOEFFLER JP AND SIDDIQUI A. (1991). HBV X protein alters the DNA binding specificity of CREB and ATF-2 by protein-protein interactions. *Science*, **252**, 842–844.

MARTIN DW, MUNOZ RM, SUBLER MA AND DEB S. (1993). p53 binds to the TATA-binding protein-TATA complex. *J. Biol. Chem.*, **268**, 13062–13067.

MERCER WE, SHIELDS MT, AMIN M, SAUVE GJ, APPELLA E, ROMANO JW AND ULLRICH SJ. (1990). Negative growth regulation in a glioblastoma tumor cell line that conditionally expresses human wild-type p53. *Proc. Natl Acad. Sci. USA*, **87**, 6166–6170.

NATIONAL RESEARCH COUNCIL. (1994). Assessment of Toxicology. In *Science and Judgement in Risk Assessment*, National Academy of Sciences (ed.) pp. 56–67. National Academy Press: Washington, DC.

NGUYEN T, BRUNSON D, CRESPI CL, PENMAN BW, WISHNOK JS AND TANNENBAUM SR. (1992). DNA damage and mutation in human cells exposed to nitric oxide in vitro. *Biochemistry*, **285**, 1173–1180.

PARK M AND VANDE WOUDE GF. (1989). Principles of molecular cell biology of cancer: oncogenes. In *Cancer: Principles & Practice of Oncology*. 3rd edn, DeVita VT, Jr., Hellman S and Rosenberg SA. (eds) pp. 45–66. JB Lippincott: New York.

PATERLINI P, POUSSIN K, KEW M, FRANCO D AND BRECHOT C. (1995). Selective accumulation of the X transcript of hepatitis B virus in patients negative for hepatitis B surface antigen with hepatocellular carcinoma. *Hepatology*, **21**, 313–321.

PERERA FP AND SANTELLA R. (1993). Carcinogenesis. In *Molecular Epidemiology: Principles and Practices*, Schulte P and Perera FP. (eds) pp. 277–300. Academic Press: New York.

PONCHEL F, PUISIEUX A, TABONE E, MICHOT JP, FROSCHL G, MOREL AP, FREBOURG T, FONTANIERE B, OBERHAMMER F AND OZTURK M. (1994). Hepatocarcinoma-specific mutant p53-249ser induces mitotic activity but has no effect on transforming growth factor beta 1-mediated apoptosis. *Cancer Res.*, **54**, 2064–2068.

PRIVES C. (1994). How loops, beta sheets, and alpha helices help us to understand p53. *Cell*, **78**, 543–546.

RAYCROFT L, WU H AND LOZANO G. (1990). Transcriptional activation by wild-type but not transforming mutants of the p53 anti-oncogene. *Science*, **249**, 1049–1051.

RIPLEY LS. (1990). Frameshift mutation: determinants of specificity. *Annu. Rev Genet.*, **24**, 189–213.

SAVITSKY K, BAR-SHIRA A, GILAD S, ROTMAN G, ZIV Y, VANAGAITE L, TAGLE DA, SMITH S, UZIEL T, SFEZ S, ASHKENAZI M, PECKER I, FRYDMAN M, HARNIK R, PATANJALI SR, SIMMONS A, CLINES GA, SARTIEL A, GATTI RA, CHESSA L, SANAI O, LAVIN MF, JASPERS NG, MALCOLM A, TAYLOR R, ARLETT CF, MIKI T, WEISSMAN SM, LOVETT M, COLLINS FS AND SHILOH Y. (1995). A single Ataxia-Telangiectasia gene with a product similar to Pi-3 kinase. *Science*, **268**, 1749–1753.

SCORSONE KA, ZHOU YZ, BUTEL JS AND SLAGLE BL. (1992). p53 mutations cluster at codon 249 in hepatitis B virus-positive hepatocellular carcinomas from China. *Cancer Res.*, **52**, 1635–1638.

SETO E, USHEVA A, ZAMBETTI GP, MOMAND J, HORIKOSHI N, WEINMANN R, LEVINE AJ AND SHENK T. (1992). Wild-type p53 binds to the TATA-binding protein and represses transcription. *Proc. Natl Acad. Sci. USA*, **89**, 12028–12032.

SHIELDS PG AND HARRIS CC. (1991). Molecular epidemiology and the genetics of environmental cancer. *JAMA*, **266**, 681–687.

SHIRAKATA Y, KAWADA M, FUJIKI Y, SANO H, ODA M, YAGINUMA K, KOBAYASHI M AND KOIKE K. (1989). The X gene of hepatitis B virus induced growth stimulation and tumorigenic transformation of mouse NIH3T3 cells. *Jpn. J. Cancer Res.*, **80**, 617–621.

SOUSSI T, CARON DE FROMENTEL C AND MAY P. (1990). Structural aspects of the p53 protein in relation to gene evolution. *Oncogene*, **5**, 945–952.

SOUSSI T, LEGROS Y, LUBIN R, ORY K AND SCHLICHTHOLZ B. (1994). Multifactorial analysis of p53 alteration in human cancer: a review. *Int. J. Cancer*, **57**, 1–9.

SPANDAU DF AND LEE CH. (1988). Trans-activation of viral enhancers by the hepatitis B virus X protein. *J. Virol.*, **62**, 427–434.

THUT CJ, CHEN J-L, KLEMM R AND TJIAN R. (1995). p53 transcriptional activation mediated by coactivators TAFii40 and TAFii60. *Science*, **267**, 100–104.

TORNALETTI S AND PFEIFER GP. (1994). Slow repair of pyrimidine dimers at p53 mutation hotspots in skin cancer. *Science*, **263**, 1436–1438.

TRUANT R, XIAO H, INGLES CJ AND GREENBLATT J. (1993). Direct interaction between the transcriptional activation domain of human p53 and the TATA box-binding protein. *J. Biol. Chem.*, **268**, 2284–2287.

TWU JS AND SCHLOEMER RH.. (1987). Transcriptional trans-activating function of hepatitis B virus. *J. Virol.*, **61**, 3448–3453.

UEDA H, ULLRICH SJ, GANGEMI JD, KAPPEL CA, NGO L, FEITELSON MA AND JAY G. (1995). Functional inactivation but not structural mutation of p53 causes liver cancer. *Nature Genet.*, **9**, 41–47.

UNSAL H, YAKICIER C, MARCAIS C, KEW M, VOLKMANN M, ZENTGRAF H, ISSELBACHER KJ AND OZTURK M. (1994). Genetic heterogeneity of hepatocellular carcinoma. *Proc. Natl Acad. Sci USA*, **91**, 822–826.

VOGELSTEIN B AND KINZLER KW. (1992*a*). p53 function and dysfunction. *Cell*, **70**, 523–526.

VOGELSTEIN B AND KINZLER KW. (1992*b*). Carcinogens leave fingerprints. *Nature*, **355**, 209–210.

WANG XW, FORRESTER K, YEH H, FEITELSON MA, GU JR AND HARRIS CC. (1994). Hepatitis B virus X protein inhibits p53 sequence-specific DNA binding, transcriptional activity and association with transcription factor ERCC3. *Proc. Natl Acad. Sci. USA*, **91**, 2230–2234.

WANG XW, YEH H, SCHAEFFER L, ROY R, MONCOLLIN V, EGLY JM, WANG Z, FRIEDBERG EC, EVANS MK, TAFFE BG, BOHR VA, HOEIJMAKERS JH, FORRESTER K AND HARRIS CC. (1995). p53 Modulation of TFIIH-associated nucleotide excision repair activity. *Nature Genet.*, **10**, 188–195.

WANG XW, GIBSON MK, VERMEULEN W, YEH H, FORRESTER K, STURZBACHER H-W, HOEIJMAKERS JHJ AND HARRIS CC. (1995). Abrogation of p53-induced apoptosis by the hepatitis B virus X gene. *Cancer Res.* (in press).

WINK DA, KASPRZAK KS, MARAGOS CM, ELESPURU RK, MISRA M, DUNAMS TM, CEBULA TA, KOCH WH, ANDREWS AW, ALLEN JS AND ET AL. (1991). DNA deaminating ability and genotoxicity of nitric oxide and its progenitors. *Science*, **254**, 1001–1003.

ZIEGLER A, JONASON AS, LEFFELL DJ, SIMON JA, SHARMA HW, KIMMELMAN J, REMINGTON L, JACKS T AND BRASH DE. (1994). Sunburn and p53 in the onset of skin cancer. *Nature*, **372**, 773–776.